# “Academia da Saúde” program: mapping evidence from the largest health promotion community program in Brazil

**DOI:** 10.3389/fpubh.2023.1227899

**Published:** 2023-07-20

**Authors:** Diego Augusto Santos Silva, Tiago Rodrigues de Lima, Letícia Gonçalves

**Affiliations:** Sports Center, Department of Physical Education, Federal University of Santa Catarina, Florianopolis, Brazil

**Keywords:** public health, nutrition, health promotion, physical activity, population health

## Abstract

The aim of this scoping review was to map the literature related to the “Academia da Saúde” Program, including the objective and rationale of the studies, activities carried out in the program’s centers, as well as the actors involved in these actions. The search for evidence was conducted in the MEDLINE, LILACS, Web of Science, Scopus, COCHRANE, and SciELO databases. Additional evidence was investigated in the Catalog of Theses and Dissertations of the Coordination of Improvement of Higher Education Personnel (CAPES-Brazil) and in the Brazilian Digital Library of Theses and Dissertations, in addition to manual searches in the references of the studies/documents. Out of 642 initial records, the information synthesis was composed of 74 studies/documents (*n* = 54; 73.0% scientific articles, *n* = 48; 64.9% with cross-sectional design, *n* = 45; 60.8% quantitative analysis). Nutrition (*n* = 24; 32.2%) and evaluation of the Program (*n* = 27; 36.5%) were the main themes analyzed. Regarding the participant/object analyzed in each study, users (*n* = 39; 52.6%) were the main actors investigated. Future studies should consider investigating the effectiveness of the actions developed in the program centers, especially physical activity and healthy eating practices.

## 1. Introduction

Non-communicable diseases (NCDs) are recognized as multifactorial diseases and they are associated with modifiable risk factors (1), including smoking, excessive alcohol consumption, inadequate food intake, and physical inactivity (2). In Brazil, NCDs (i.e., cardiovascular diseases, cancer, chronic respiratory diseases, and diabetes mellitus) are responsible for approximately 72% of all deaths (3, 4), disproportionately affecting vulnerable groups (5), such as the older adults, individuals with low income, low education, and those with limited access to public health care services (6).

In Brazil, actions and programs derived from public policies have been implemented with the objective of reducing NCDs. Among the nationally conducted actions, the Strategic Action Plan for Coping with NCDs 2011–2022 (2), developed by the Brazilian Ministry of Health, stands out. This action aims to develop and implement public policies for the prevention and control of morbidity and risk factors through the strengthening of health services (1, 2). The Strategic Action Plan for Coping with NCDs 2011–2022 is based on three main pillars: (1) surveillance, information, evaluation, and monitoring; (2) health promotion; (3) comprehensive care (1–3). Regarding health promotion, the “Academia da Saúde” Program (free translation in English: *Health Academy Program*) deserves attention, as the actions carried out in the program strengthen health promotion activities in Primary Care (6).

The “Academia da Saúde” Program is a continuous health promotion initiative, involving the participation and financing of municipalities, states, and the federal government (7), implemented throughout the entire Brazilian territory, making it the most comprehensive health promotion program in the country. This program includes axes/activities related to bodily practices and physical activities, production of care and healthy lifestyles, promotion of healthy eating, integrative and complementary practices, artistic and cultural practices, health education, planning and management, and community mobilization (8). Such activities are guided and supervised by qualified professionals and take place in public spaces specifically implemented (poles) for this purpose (9–11). In 2018, there were a total of 2,300 poles of the Program across the Brazil (12).

Previous studies showed that the “Academia da Saúde” Program is in line with the principles of the Brazilian health system (11), contributes to the expansion of community health promotion programs (11) and provides access to health promotion actions for the less privileged population (13). However, information regarding the description and rationale of the actions taken, impact of the activities on health indicators, and/or advances in terms of public health resulting from the “Academia da Saúde” Program are not known. Identifying and summarizing evidence related to the “Academia da Saúde” Program can contribute to the recognition of successful activities developed by the program and, thus, favor the expansion and extrapolation of such practices beyond the community or primary health care context. Preliminary searches were conducted in MEDLINE, LILACS, Web of Science, Scopus, COCHRANE, and SciELO databases; however, no scope reviews or systematic reviews with the same objective as the study were identified. While previously published review studies aimed to summarize actions developed in health promotion programs in Brazil, such as the “Academia da Saúde” Program, the present study contributes to the literature by providing information regarding the objective and rationale for conducting the original studies carried out in the centers (i.e., poles) of the “Academia da Saúde” Program.

The present scope review aimed to map the evidence related to the “Academia da Saúde” Program, including the objective and rationale of the studies, activities carried out in the program’s poles, as well as the actors involved in these actions.

## 2. Methods

### 2.1. Protocol

The present scoping review study was established based on the following research question: What evidence underlies the different stages of the “Academia da Saúde” Program? The research protocol was registered on the Open Science Framework–OSF,^1^ an online science sharing platform, and was developed based on the guidelines of the Preferred Reporting Items for Systematic Reviews and Meta-Analyzes extension for Scoping Reviews - PRISMA-ScR (14) and Joanna Briggs Institute–JBI guidelines (15). The review structure followed the JBI recommended steps: (i) identification of the research question; (ii) evidence tracking related to the topic; (iii) evidence selection; (iv) in-formation analysis; (v) grouping, synthesis, and presentation of information/data.

### 2.2. Eligibility criteria of the study

As inclusion criteria, original quantitative or qualitative articles with cross-sectional, longitudinal, case–control, ecological, intervention, or bibliographic/documentary designs were sought. In addition, Thesis and Dissertation documents whose objective was analogous to that of this review (i.e., to present evidence related to the design, development, implementation, and conduct of activities carried out in Program) were also included. Furthermore, sources of evidence published in Portuguese, English, and Spanish were considered. Additionally, there were no restrictions on the years of data collection and publication, topics addressed, or strategies adopted in data collection and analysis. Evidence derived from review studies, monographs, thesis, dissertations, non-scientific/non-technical texts from the internet, editorials, essays, or studies not available for full access in the investigated data sources were excluded.

### 2.3. Selection of evidence sources

Two authors independently examined each database to identify potential studies/documents. After extracting the studies/documents from the databases, du-plicate information was excluded, and then studies/documents that did not meet the inclusion criteria previously reported were excluded after reading the titles and abstracts. Subsequently, these selected studies/documents were read in full to select the texts that would be included. Disagreements between the two reviewers were resolved through a consensus meeting. A third reviewer was consulted when disagreements/doubts regarding the studies/documents were not resolved by the reviewers. The EndNote® soft-ware version X6–(Thomson ISI ResearchSoft–Clarivate Analytics, Philadelphia, United States, 2010) was used to manage the studies/documents verified, as the functions provided by the software allow the identification and exclusion of duplicate studies, and the organization of information from each database.

### 2.4. Search strategies

The search for available evidence related to the theme was conducted during February 2022 in the MEDLINE, LILACS, Web of Science, Scopus, COCHRANE, and SciELO databases, based on the search strategy applied in MEDLINE: [Health gym(Text Word)] OR [Health academy(Text Word)] OR [Academia da Saúde Program(Text Word)] OR [Health Academy Program(Text Word)] AND [Brazil(Text Word)]. Additional evidence was investigated in the Catalog of Theses and Dissertations of the Coordination for the Improvement of Higher Education Personnel (CAPES)^2^ and in the Brazilian Digital Library of Theses and Dissertations (BDTD).^3^ As an additional resource, manual searches were conducted in the references of the studies/documents. Additional information regarding the evidence sources can be found in Supplementary Table S1.

### 2.5. Extraction and synthesis of information

Two authors conducted the extraction and synthesis of information derived from the included studies/documents, using a standardized data spreadsheet. The extracted descriptive information included characteristics of the studies/documents (place of study/document realization, publication year, number of participants, nature of the studies/documents, study/document design, and information analysis). Given that the present scope review aimed to map the literature on the Program regardless of quality, the risk of bias was not assessed (16).

The descriptive synthesis of the studies/documents included in this study followed the guidelines suggested by JBI (15), which employ three elements to guide the extraction of information: Population–information related to government agents, Program coordinators, healthcare professionals, and users; Concept–evidence related to the conception, development, implementation, and execution of activities; Context–“Academia da Saúde” Program.

After extracting the descriptive information from the included studies/documents and considering the diversity of investigated topics, it was decided to synthesize such information based on how the object of investigation of each analyzed study was related to the “Academia da Saúde” Program. In this context, seven major categories were defined: (i) information from participants was used in the investigation of different objectives and related aspects; (ii) methodological description, evaluation, and/or results of intervention programs conducted with users; (iii) spatial distribution of program units and their relationship with the food environment; (iv) quantitative and/or qualitative synthesis of actions performed, health indicators, and socioeconomic descriptors related to the program; (v) understanding and description of meanings, knowledge involved, and foundations required for the conception and/or execution of activities carried out in the program; (vi) understanding of users’ perspectives on the program and activities developed; (vii) evaluability and/or implementation of the program.

In addition to the seven major categories adopted to synthesize the diversity of investigated topics, we decided to synthesize the information considering the topics of the studies/documents according to the analyzed objects. The topics from which the information was extracted are as follows: (1) nutrition; (2) nutrition and physical activity; (3) nutrition, physical activity, and anthropometric variables; (4) nutrition and anthropometric profile; (5) physical activity; (6) program evaluation; (7) local-level instrument evaluation (i.e., assessing the validity of the investigation methods used by the study); (8) local-level program description and evaluation; (9) methodological study; (10) healthy lifestyle; (11) intervention evaluation conducted within the program context; (12) reduction of healthcare expenditures; (13) health education. Regarding the object of investigation in each analyzed study/document, eight distinct categories were identified: (a) users; (b) managers; (c) Physical Education professionals; (d) territory; (e) Health Academy Program; (f) users and territory; (g) managers and professionals; (h) managers, professionals, and users.

## 3. Results

**Figure 1:**
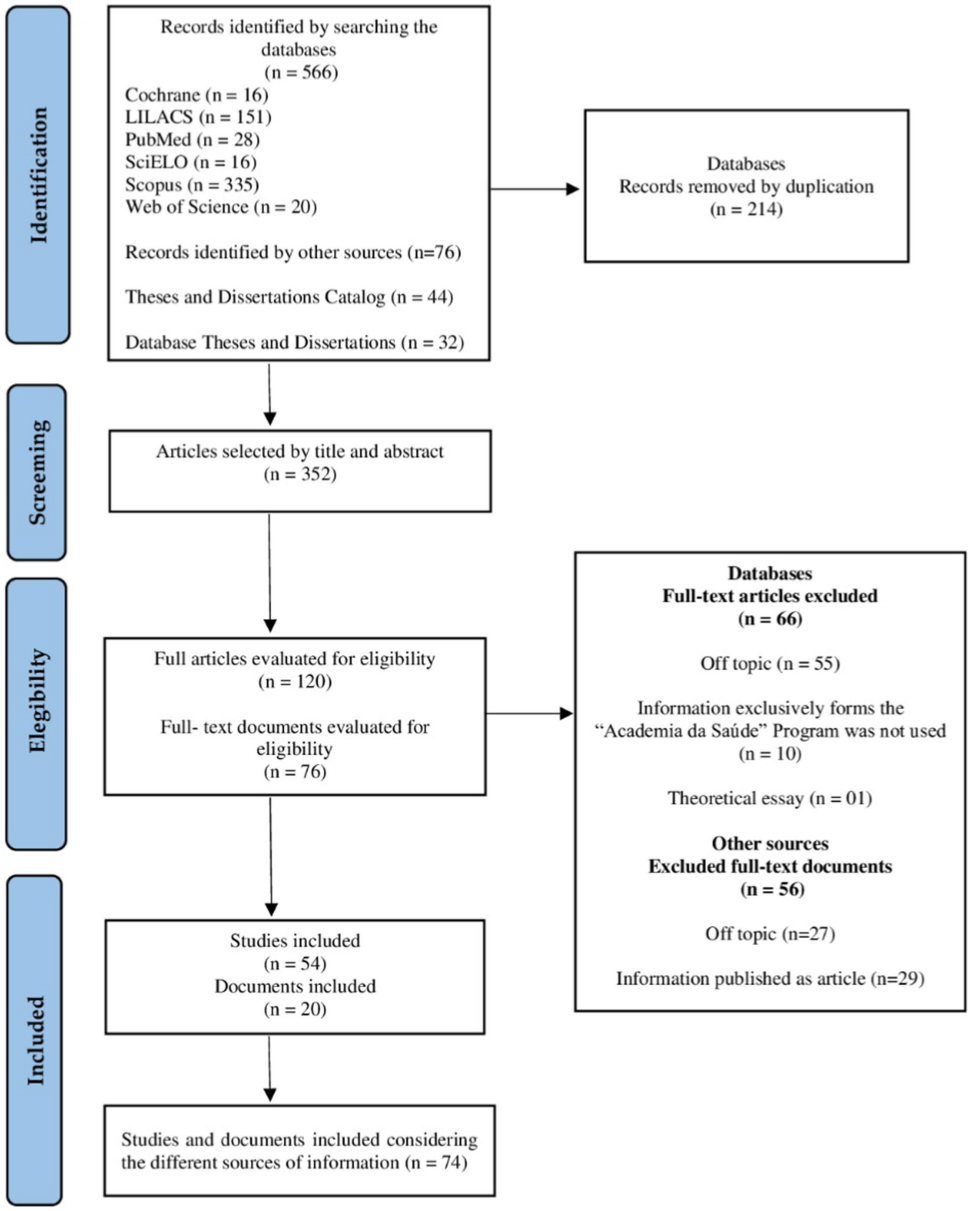
Flowchart of searches for documents in this scoping review.

The database search resulted in a total of 566 identified titles. After removing duplicates (*n* = 214), screening title and abstract (*n* = 232), and analyzing full-text articles (*n* = 66), 54 studies were included for information synthesis. In addition to the database search, the Catalog of Theses and Dissertations, and the BDTD were investigated to identify theses and dissertations related to the topic of interest in this review. Out of the initially identified 76 records, 27 documents were excluded for not meeting the inclusion criteria, and 29 were excluded as the analyzed information was subsequently published as a scientific article, resulting in a total of 20 documents. Therefore, a total of 74 studies/documents were included considering the different sources of information (Figure 1).

The descriptive characteristics of the included studies/documents are described in Supplementary Table S2. Briefly, the analyzed information was mostly derived from studies conducted in the state of Minas Gerais (*n* = 40; 54.4%), with a growing number of publications from the biennium 2016–2017 (*n* = 20; 86.5%), and involving up to 99 participants (*n* = 20; 31.0%). Regarding the nature, design, and analysis of the information derived from the studies/documents, 73.0% (*n* = 54) were scientific articles, 64.9% were analyzed in a cross-sectional design, and 60.8% (*n* = 45) were conducted in a quantitative way, respectively.

Regarding the purpose of the included studies/documents, seven domains were identified (Table 1). Approximately one in three studies (35.1%) used information from users to investigate the association between various variables. Ten studies (13.5%) aimed to describe the methodology used, evaluate and/or present results of intervention programs conducted with users, but not related to the program itself. Analyzing the spatial distribution of program units and their relationship with the food environment was the objective of three studies (4.0%). In addition, seven studies aimed to summarize quantitatively and/or qualitatively the actions conducted, health indicators, and socioeconomic descriptors related to the program (9.5%). In addition to the aforementioned domains, understanding and describing the meanings, knowledge involved, and the necessary basis for the design and/or implementation of program activities directed the analyzes of 13 studies/documents (17.6%), while understanding users’ perceptions of the program and activities (*n* = 02; 2.7%) and evaluating and/or implementing the program (*n* = 13; 17.6%) were the basis for investigations conducted in the remaining studies/documents analyzed.

**Table 1:** Description of the objectives investigated in each study, according to the “Academia da Saúde” Program.

Domain number	Domains	*n*	%	References
i	Information from the participants of the “Academia da Saúde” Program was used in the investigation of different objectives and related aspects	26	35.1	(17–42)
ii	Methodological description, evaluation and/or results of intervention programs conducted with participants of the “Academia da Saúde” Program	10	13.5	(43–52)
iii	Spatial distribution of the units of the “Academia da Saúde” Program and the relationship with the food environment	03	4.0	(53–55)
iv	Quantitative and/or qualitative summary of the actions carried out, health indicators and socioeconomic descriptors related to the “Academia da Saúde” Program	07	9.5	(56–62)
v	Understanding and description of the meanings, knowledge involved and necessary background for the design and/or conduct of activities carried out in the “Academia da Saúde” Program	13	17.6	(13, 63–74)
vi	Understanding of users about the “Academia da Saúde” Program and activities developed	02	2.7	(75, 76)
vii	Evaluability and/or implementation of the “Academia da Saúde” Program	13	17.6	(77–89)

Thirteen different topics were investigated by the studies/documents included in this review. The majority of studies had Nutrition (*n* = 24; 32.2%) and Evaluation of the Program (*n* = 27; 36.5%) as their main themes. Other investigated topics were related to Nutrition and Physical Activity (*n* = 2; 2.7%), Nutrition, Physical Activity and Anthropometric variables (*n* = 2; 2.7%), Nutrition and Anthropometric Profile (*n* = 3; 4.1%), and Physical Activity (*n* = 7; 9.4%). Other topics were also investigated, including Instrument evaluation at local level (*n* = 1; 1.4%), Health Education (*n* = 1; 1.4%), Description and Evaluation of the Program at the local level (*n* = 2; 2.7%), Methodological study (*n* = 1; 1.4%), Healthy lifestyle (*n* = 1; 1.4%), Evaluation of a Program conducted within the context of the “Academia da Saúde” Program (*n* = 2; 2.7%), and Reduction of health expenses (*n* = 1; 1.4%; Table 2).

**Table 2:** Topics investigated, according to the participant/investigation object of the included studies.

Investigated topic	Participant/investigated object	Number (percentage) referring to the analyzed topic	Number (percentage) referring to the total of the table	Reference
Nutrition			24 (32.2)	(17–33, 41, 43–46, 53, 54)
	Users	18 (75.0)		(17–29, 41, 43–46)
	Territory	01 (4.2)		(53)
	Users and Territory	05 (20.8)		(30–33, 54)
	Nutrition and physical activity			02 (2.7)	(34, 56)
Users		01 (50.0)		(34)
Managers		01 (50.0)		(56)
Nutrition, physical activity and anthropometric variables			02 (2.7)	(47, 48)
	Users	02 (100.0)		(47, 48)
Nutrition and anthropometric profile			03 (4.1)	(35, 36, 49)
	Users	03 (100.0)		(35, 36, 49)
Physical activity			07 (9.4)	(40, 57, 58, 63–65, 75)
	Users	05 (71.4)		(40, 57, 58, 65, 75)
	Physical Education Professionals	02 (28.6)		(63, 64)
Evaluation of the program			27 (36.5)	(13, 37, 55, 59–61, 66–74, 77–86, 88, 89)
	Users	02 (7.4)		(37, 61)
	Users and Territory	01 (3.7)		(55)
	Managers	04 (14.8)		(59, 81–83)
	Physical Education Professionals	03 (11.1)		(13, 70, 71)
	“Academia da Saúde” Program	08 (29.7)		(66–69, 77–79, 89)
	Managers and Professionals	03 (11.1)		(74, 80, 88)
	Managers, Professionals and Users	06 (22.2)		(60, 72, 73, 84–86)
	“Academia da Saúde” Program	01 (100.0)		(38)
Evaluation of a local instrument			01 (1.4)	(38)
	“Academia da Saúde” Program	01 (100.0)		
Health education			01 (1.4)	(76)
	Users	01 (100.0)		(76)
Description and evaluation of the program at the local level			02 (2.7)	(62, 87)
	Users	02 (100.0)		(62, 87)
Methodological study			01 (1.4)	(50)
	Users	01 (100.0)		(50)
Healthy lifestyle			01 (1.4)	(39)
	Users	01 (100.0)		(39)
Evaluation of a program conducted within the context of the “Academia da Saúde” Program			02 (2.7)	(51, 52)
	Users	02 (100.0)		(51, 52)
Reduction of health expenses			01 (1.4)	(42)
	Users	01 (100.0)		(42)

Regarding the participant/object analyzed in each study, eight categories were investigated: (i) users (*n* = 39; 52.6%); (ii) managers (*n* = 6; 6.8%); (iii) Physical Education professionals (*n* = 5; 6.8%); (iv) territory (*n* = 1; 1.4%); (v) “Academia da Saúde” Program (*n* = 9; 12.2%); (vi) users and territory (*n* = 6; 8.1%); (vii) managers and professionals (*n* = 3; 4.0%); (viii) managers, professionals, and users (*n* = 6; 8.1%; Table 2).

Specific information on the studies/documents included (i.e., object of investigation, relation of the study to the review objective, topic analyzed, strategy adopted to conduct the activities, and results identified) can be found in Supplementary Table S2.

## 4. Discussion

As previously evidenced by studies with similar objectives and design to the present scoping review (11, 90), Nutrition and Program Evaluation were the most investigated topics by studies related to the “Academia da Saúde” Program, with the main focus of these studies being the program users. According to Article 70 of the Consolidation Ordinance No. 5, of September 28, 2017, nutrition is a component of the axis of actions (promotion of healthy eating) that guides the activities to be developed in the “Academia da Saúde” poles. The relevance of nutrition in the context of the “Academia da Saúde” Program is explained by the numerous themes related to the topic investigated by the studies, which focused on the consumption of fruits and vegetables (17, 26, 28, 29), dietary profile (27, 41), access to healthy food consumption (22, 23, 25, 30–33, 53, 54), nutritional knowledge (20, 21, 24), identification of barriers and facilitators for the intake of fruits and vegetables (18, 19, 40), and interventions aimed at promoting the intake of healthy foods (44–46). The relevance of investigating this topic by the included studies is even more understandable when considering that dietary intake has a direct relationship with the occurrence of NCDs (e.g., cancer, heart disease, and obesity) (91–93), as prevention and control of morbidities and risk factors associated with NCDs are the pillars of the creation of the “Academia da Saúde” Program (8).

The evidence synthesis revealed a high number of studies that aimed to evaluate the “Academia da Saúde” Program. It is important to highlight that this type of study allows the investigated information to be used as a management tool, contributing to decision-making regarding the actions carried out by the Program (94). In addition, despite investments in health promotion and prevention being often less costly compared to the expenses incurred with the treatment of NCDs (95), budgetary and human resource constraints available for complementary programs to primary health care actions indicate the need for evaluation of activities related to the “Academia da Saúde” Program, which may justify the high number of evidence available in the literature on this topic.

Although the “Academia da Saúde” Program was created with the objective of promoting physical practices, regular physical activity, healthy eating, health education, interdisciplinary monitoring, as well as contributing to the production of healthy and sustainable lifestyles and care for the general population^7^, the synthesis of information conducted in this review indicated that a large part of the studies did not address the investigation of these themes or did not report how the actions carried out in the poles translated into results for the users of the program. In this sense, in addition to systematically reporting on how activities are developed with users (e.g., type of activity, number of meetings/sessions, basis and objective of the activity carried out), it is suggested that future studies related to the “Academia da Saúde” Program aim to investigate the relationship between activities developed in the poles and the themes for which the program was created (e.g., healthy lifestyle), in order to identify successful practices and, consequently, enable their replication.

Although the extensive literature review conducted in different databases to identify the literature related to the “Academia da Saúde” Program (scientific articles, theses, and dissertations) is a strong point of the present study, some limitations need to be declared: (i) given the characteristics of the scoping review (i.e., identifying the main concepts related to a particular area of research or topic, which allows for a deeper exploration of the de-scribed information - unlike other systematic review studies, which aim to evaluate the quality of available evidence), the studies included in this review were not evaluated in terms of methodological rigor; (ii) although the strategy adopted to gather available information was comprehensive in terms of sources, it is possible that relevant studies related to the topic may not have been published in the searched databases.

We concluded that Nutrition and Evaluation of the Program were the most evaluated topics by the studies, with program users being the main actors in these investigations. Future studies should consider investigating the effectiveness of the actions developed in the program’s poles, especially physical activity and healthy eating, which are the assumptions for the creation of the “Academia da Saúde” Program.

## Author contributions

DS: conceptualization, methodology, formal analysis, visualization, and writing–original draft. TL: investigation and writing–reviewing and editing. LG: investigation and writing–reviewing and editing. All authors contributed to the article and approved the submitted version.

## Funding

DS was financed in part by the Coordenação de Aperfeiçoamento de Pessoal de Nível Superior–Brazil (CAPES)–Finance Code 001 and he is supported in part by National Council for Scientific and Technological–CNPq, Brazil (309589/2021–5; 200636/2022–7).

## Conflict of interest

The authors declare that the research was conducted in the absence of any commercial or financial relationships that could be construed as a potential conflict of interest.

## Publisher’s note

All claims expressed in this article are solely those of the authors and do not necessarily represent those of their affiliated organizations, or those of the publisher, the editors and the reviewers. Any product that may be evaluated in this article, or claim that may be made by its manufacturer, is not guaranteed or endorsed by the publisher.

## Supplementary material

The Supplementary material for this article can be found online at: https://www.frontiersin.org/articles/10.3389/fpubh.2023.1227899/full#supplementary-material

## Footnotes

^1^ https://osf.io/re75p

^2^ https://catalogodeteses.capes.gov.br/catalogo-teses/#!/

^3^ http://bdtd.ibict.br/
