# Supplementary material for: “Academia da Saúde” program: mapping evidence from the largest health promotion community program in Brazil
Source: Front Public Health. 2023 Jul 20;11:1227899. doi: 10.3389/fpubh.2023.1227899 (PMC10400361; doi:10.3389/fpubh.2023.1227899)
Supplement: Supplementary file 1 [file Data_Sheet_1.PDF]

Supplementary Material

"Academia da Saúde" Program: mapping evidence from the largest health promotion community program in Brazil

Diego Augusto Santos Silva[1]*, Tiago Rodrigues de Lima[1], Letícia Gonçalves[1]

***Correspondence:** Diego Augusto Santos Silva: diegoaugustoss@yahoo.com.br

Supplementary Table 1. Specific information on included studies (n = 74).

Research object	Reference	Relationship between the study and the purpose of the review	Strategy adopted to conduct activities	Identified results
**Topic analyzed**				
**Nutrition**				
Users, n = 18				
	[18]	To investigate the consumption of fruits and vegetables among families of users of the "Academia da Saúde" Program poles.	A semi-structured interview was used, with the script of questions divided into three thematic blocks: 1) eating habits in the family; 2) perception of fruit and vegetable consumption by the family; 3) acquisition of fruits and vegetables.	The construction of meanings for the consumption of fruits and vegetables in the investigated population occurred through the constitution of family relationships and the incorporation of the mechanistic discourse on food and health, with repercussions on the modulation of taste and food choices.
	[19]	To investigate barriers and factors that promote the consumption of fruits and vegetables among users of the "Academia da Saúde"	The interview script included questions related to eating practices, consumption and acquisition of fruits and vegetables, and distributed in the	The main barriers identified were: inadequate trade, low purchasing power, price, lack of public initiatives, lack of time, laziness, fruit being considered only a food alternative and not having

	Program.	following categories: 1) barriers to the consumption of fruits and vegetables; 2) factors promoting the consumption of fruits and vegetables.	dinner. The most cited promoting factors were: health, prevention/disease control, liking, vegetables being considered as part of the meal, creation and family origin, improvement of the financial situation, nearby trade and purchasing strategies.
[20]	Understand the elements that interfere with adherence to actions to encourage the consumption of fruits and vegetables among users of the “Academia da Saúde” Program.	The interviews were conducted with users of the “Academia da Saúde Program” who received nutritional interventions to encourage the consumption of fruits and vegetables based on the Transtheoretical Model.	The elements that facilitate adherence to actions to encourage the consumption of fruits and vegetables were the “Academia da Saúde” Program itself, the methodology used in the intervention, aspects inherent to the subject, family support, the bond with the group, with the service and with the team . The complicating elements were: domestic and/or external work, caring for others, and personal commitments – elements permeated by the aspect of the time of actions.

[21]	To assess the prevalence of inaccurate reporting of energy intake and its associated factors.	The assessment of energy intake was carried out using 24-hour food recalls and imprecise reporting using the McCrory methodology [96].	The prevalence of underreporting of food consumption was 11.9%, being more prevalent among participants with dyslipidemia and very high waist circumference, and less prevalent among adults, those satisfied with body weight and with equal or higher dietary fractionation to five meals a day.
[22]	To compare the perception and consumption of fruits and vegetables and identify the factors associated with the mistaken perception of intake.	The stages of change from the Transtheoretical Model were used to assess perceptions regarding intake, and questions adapted from national surveys were used to assess fruit and vegetable intake as separate groups. Individuals whose intake and perception were discordant were reclassified into pseudo-maintenance or non-reflective action.	Pseudo-maintenance of fruit consumption was associated with gender, education, food and nutrition security, satisfaction with weight and participation in nutritional interventions; the pseudo-maintenance of vegetable intake was associated with schooling and satisfaction with weight. Non-reflexive action was associated with age.

[23]	To identify the effects of the food environment and perceptions of self-efficacy on fruit and vegetable consumption.	Perceptions of the food environment were measured by survey, in which participants indicated how confident they were about the availability of fruits and vegetables in the food environment. Self-efficacy statements were assessed in three domains regarding perceived accessibility, time, and cooking skills.	The association of perception of the food environment with consumption of fruits and vegetables was marginally significant ($p=0.062$), while self-efficacy was more strongly associated with consumption of fruits and vegetables ($p < 0.001$). Additionally, a confidence score greater than one standard deviation was associated with a 35.1 g higher intake of fruits and vegetables.
[24]	To examine associations of individual environmental and dietary factors with fruit and vegetable consumption.	Data on the individual and food environment were obtained through: (i) face-to-face interviews; and (ii) food store audits.	Individual-level factors including age, income, food insecurity, stage of change, self-efficacy, and decision-making balance were significantly associated with fruit and vegetable intake. After controlling for individual-level characteristics, higher fruit and vegetable intake was associated with higher quality food stores.

[25]	To evaluate the nutritional status and its association with the nutritional knowledge of users assisted by the “Academia da Saúde” Program.	Nutritional status was assessed using the body mass index, and a score derived from a questionnaire containing twelve questions was used to obtain information regarding food and nutrition.	No significant associations between the analyzed parameters were found.
[44]	To investigate barriers and facilitators for participants' adherence to a nutritional intervention.	Intervention based on the Transtheoretical Model and Paulo Freire's pedagogy, and offered 12 educational activities with the aim of promoting the consumption of fruits and vegetables.	Adherence was associated with aging, being unemployed, not undergoing psychiatric treatment, body satisfaction and participation of the “Academia da Saúde” Program user for > 1 year. Qualitative analysis revealed the following facilitators for adherence: service structure, intervention methodology, building bonds between users and professionals, family support and associated aspects. Adherence barriers included work, self-care and care for others.

[26]	To analyze the quantity and diversity of consumption of fruits and vegetables, as well as their relationship with characteristics of acquisition and the consumer's food environment.	The consumption of fruits and vegetables was investigated using the Food Frequency Questionnaire. Users were also asked about the frequency, place of purchase and availability of food at home. To assess the consumer's food environment, an audit was carried out of commercial establishments within a radius of 1.6 km around the sampled units of the program.	Knowledge about food crops, increased monthly availability of fruit at home, greater variety of fruit and quality of vegetables in commercial establishments could improve the quantitative consumption of fruits and vegetables, while a greater variety of fruits could increase the diversity of consumption.
[27]	To identify the factors associated with the inadequate consumption of fruits and vegetables in users of the “Academia da Saúde” Program.	Inadequate consumption of fruits and vegetables were investigated as an outcome. Sociodemographic variables, health issues, anthropometry and fruit and vegetable purchase profile were analyzed as	Inadequate consumption of fruits and vegetables was being male, age group, evaluating quality of life as poor and not having knowledge about the food season. Factors associated only with inadequate fruit consumption, not having diabetes mellitus and having the habit of smoking.

		explanatory factors.	Having up to four years of schooling was associated only with inadequate consumption of vegetables.
[45]	To describe the effect of pseudomaintenance on progression through the stages of behavior change for fruit and vegetable intake among users of the “Academia da Saúde” Program after intervention based on the Transtheoretical model.	The stages of change for fruit and vegetable intake were evaluated using previously used algorithms [97].	Users in pseudomaintenance at baseline were more likely to progress to higher stages with regard to fruit consumption. No difference was seen in the progression to vegetable consumption.
[42]	To investigate the food consumption of participants in the Academia da Saúde Program, according to the NOVA classification, and its relationship with the	The receipt of nutritional guidance was self-reported. Food consumption was classified according to the NOVA (in natura/minimally processed foods; processed culinary ingredients; processed	The highest energy contribution to the diet came from in natura/minimally processed foods (56.6%), followed by ultra processed foods (27.7%). A lower percentage of energy derived from culinary ingredients was found among those who reported receiving nutritional

	report of receiving nutritional guidance.	and ultra-processed) and estimated in total calories and percentage of energy. Sociodemographic data were investigated; of health; food consumption, obtained by averaging two 24-hour Food Recalls; and anthropometry.	guidance (5.2%; 95%CI: 4.9-5.5 vs. 4.5%; 95%CI: 4.3-4.7 ; p < 0.01), with no significant differences for the other food groups.
[46]	To describe the effectiveness of an intervention based on the Transtheoretical model with the aim of increasing fruit and vegetable intake according to the initial perception of intake adequacy.	Pseudo-maintenance was identified when participants had insufficient intake of fruits and vegetables and considered their intake adequate. The intervention group received an intervention based on the Transtheoretical model and the control group received usual care.	Pseudomaintenance participants were partially responsive to the intervention and better fruit intake at follow-up.
[28]	To describe both the diet and nutrient intake,	Food and nutritional intake was assessed using the average of	Users in the third tertile with regard to time in the "Academia da Saúde"

	according to the time of participation in the "Academia da Saúde" Program.	two 24-hour recalls.	Program (24.4 to 61.6 months) had a lower intake of energy, lipids and ultra-processed foods and more culinary preparations, compared to the others. Users in the second (10·1–24·3 months) and third tertiles regarding time in the "Academia da Saúde" Program had a higher intake of carbohydrates, calcium and vitamin C compared to those in the first tertile (0–10 months).
[29]	To investigate the association between different generations and consumption of fruits and vegetables among users of the "Academia da Saúde" Program.	Consumption of fruits and vegetables was investigated using a questionnaire. The generations were classified according to the year of birth provided by the participants in traditionalists (1934 - 1945), baby boomer (BB; 1946 - 1964), generation X (GX; 1965 - 1980) and generation Y (GY; 1981 -	There were less chances of regular consumption of fruits and fruits + vegetables for all generations compared to traditionalists. With regard to adequate consumption, there was a lower chance of consuming fruits + vegetables only for individuals in the GY group and fruit + vegetables for the GX and GY groups.

		1993).	
[47]	To evaluate the effectiveness of a nutritional intervention to promote fruit and vegetable intake.	All evaluated participated in physical exercise sessions. The intervention to promote fruit and vegetable intake was based on Paulo Freire's transtheoretical model and pedagogy. Interventions included group educational sessions, motivational cards and informational materials. The primary outcome was a change in fruit and vegetable intake, and secondary outcomes included stages of change, self-efficacy, decision-making balance, and fruit and vegetable knowledge. All data were collected in person, and fruit and vegetable consumption was assessed using	The nutritional intervention was effective in increasing fruit and vegetable intake and fruit consumption among individuals with lower initial consumption and in maintaining fruit and vegetable consumption among those who reported consuming fruits and vegetables as recommended (400 g/ d).

		a questionnaire.	
[30]	To verify the influence of food insecurity on the consumption of fruits and vegetables.	The influence of food insecurity on fruit consumption was investigated by using a questionnaire.	Approximately 41.0% (n=367) of the families were in a situation of food insecurity. Food insecurity negatively influenced fruit and vegetable consumption.
<u>Territory, n = 01</u>			
[54]	Spatial analysis of the distribution and access to commercial fruit and vegetable establishments in the territory of a representative sample of poles of the “Academia da Saúde” Program.	Establishments contained within buffers with a radius of 1,600 meters from 18 randomly sampled poles were evaluated. The quality of access to fruits and vegetables was evaluated by the Access to Food in Establishments Index (HFSI), composed of variables of availability, variety and advertising of fruits and vegetables and ultra-processed foods.	Limited access to commercial establishments that offer quality fruits and vegetables in the territory of the “Academia da Saúde” Program was verified.

Users and Territory, n = 05

[55]	To describe the environmental conditions and their relationship with the consumption of fruits and vegetables among users of the “Academia da Saúde” Program.	Food stores within a radius of 1600m of proximity to 18 Poles of the Academia da Saúde Program were investigated. Community (density, proximity, and type) and consumer variables (sectional location of fruits and vegetables; availability, quality, variety, price, and advertising of fruits and vegetables and ultra-processed foods) were measured by direct observation, while aggregated data from users (income and consumption of fruits and vegetables) were obtained by interview.	The average consumption of fruits and vegetables was higher in neighborhoods with higher income and concentration of food stores and better access to healthy foods. Locations with low fruit and vegetable consumption had the highest number of stores with little access to healthy foods.
[31]	To investigate whether access to healthy foods	Socioeconomic status was investigated through face-to-face	A 1% increase in the availability of specialty fruit and vegetable markets or

	varies according to the type of store and the socioeconomic level of users of the "Academia da Saúde" Program.	interviews. Food stores were audited through direct observation. Variables included the community nutrition environment (type and location) and the consumer nutrition environment (health food store index, involving variables such as availability, variety, and advertising of healthy and unhealthy products).	outdoor food markets and supermarkets increased the health food store index values by 0.12 and 0.07, respectively.
[32]	To investigate the association between aspects of the consumer's food environment in food stores that sell fruits and vegetables and the incidence of overweight among users of the "Academia da Saúde"	Users of "Academia da Saúde" Program Poles and multiple aspects of the consumer's food environment (availability, ability, diversity, variety, quality, advertising and price) related to fruits and vegetables and ultra-processed foods were examined.	Over 70% of stores had adequate diversity and variety of fruits and vegetables; As for quality, only 24.5% of the stores had inadequate fruit quality and 39.6% inadequate vegetable quality. Ultra-processed foods were present in 60.6% of fruit and vegetable stores. The results indicated a high prevalence of overweight (62.6%) in the participants of

	Program.		the health promotion service and the multilevel models revealed an association with the variety of vegetables in the stores (0.99; 95% confidence interval, 0.97 -0.99; P < 0.05).
[33]	The aim of the study was to investigate the availability and advertising of food in food establishments.	Availability of fruits and vegetables, availability of ultra-processed foods, and food advertising were compared across categories of food establishments (large supermarket chains, specialty fruit and vegetable markets, and local grocers, convenience stores, or bakeries.	Food advertising was absent in 59.8% of food establishments, 19.6% only advertised fruit and vegetables, and 17.4% only advertised ultra-processed foods. Greater fruit and vegetable availability was observed within specialized fruit and vegetable markets and large supermarket chains than local grocery stores, convenience stores or bakeries. Advertising for fruit and vegetables was more common in specialist fruit and vegetable markets. However, large supermarket chains and local grocery stores, convenience stores or bakeries contained more frequent

			isolated advertising of ultra-processed foods: 38.3 and 35.2%, respectively. Thus, the availability and advertising of food in food establishments in primary care services are different according to the type of food establishment.
[34]	To examine associations between economic residential segregation and the prevalence of healthy and unhealthy eating markers.	The prevalence of dietary markers and their 95% CI were estimated according to residential economic segregation: high (more segregated); medium (integrated) and low (less segregated or integrated). Segregation was measured in the census tract and evaluated using the local Getis–Ord Gj*, a statistic based on the proportion of heads of household in a neighborhood with a monthly	Economic residential segregation was associated with markers of healthy eating, even after adjusting for individual-level factors and perceived eating environment.

		income of 0 to 3 minimum wages.	
**Nutrition and Physical Activity**			
Users, n = 01			
[35]	To investigate factors associated with receiving counseling on nutrition and physical activity.	Sociodemographic and anthropometric data were collected on food consumption, use of services and health conditions and reports of receiving counseling from users of the “Academia da Saúde” Program Poles.	The following variables were associated with receiving counseling ($p<0.05$): having high blood pressure, hypercholesterolemia, diabetes, being overweight; use medication and participate in the Academia da Saúde Program.
Managers, n = 01			
[57]	The objectives of the present study were to describe the prevalence of actions to promote physical activity and healthy eating and the characteristics of the family health strategy	A telephone interview was conducted with health managers from municipalities throughout Brazil that received funds for the development of the “Academia da Saúde” Program.	The prevalence of physical activity actions (91.5%, $p < 0.001$) and healthy eating (88.2%, $p = 0.006$) was higher in municipalities that had NASF compared to those that did not.

	in Brazilian municipalities that received resources for the development of the “Academia da Saúde” Program, and to verify the prevalence of these actions according to the presence of NASF and physical education professionals and nutritionists in the teams.		
	**Nutrition, Physical Activity and Anthropometric Variables**		
Users, n = 02			
[48]	To evaluate the effectiveness of the VAMOS (Active Life Improving Health) strategy in improving physical activity, eating habits and anthropometric variables of	Participants in the intervention (IG) and control (CG) groups participated in physical activity classes taught in the “Academia da Saúde” Program and those in GI also participated in the VAMOS strategy for 12 weeks	The intention-to-treat analysis revealed that IG participants increased the daily time of moderate-vigorous physical activity and the frequency of raw vegetable intake, while the intake of ultra-processed foods was reduced. Weight loss among participants who

	users of the “Academia da Saúde” Program.	(based on social cognitive theory). The main behavioral constructs addressed were self-efficacy, goal setting, self-monitoring, identification of supports and social barriers, and solutions for identified barriers. Physical activity was measured with accelerometers and nutritional status was assessed using questionnaires about eating habits and anthropometric measurements.	were classified as overweight/obese at baseline was observed in the intervention group compared with the control.
[49]	To evaluate the maintenance of the beneficial effects of the Active Life Improvement in Health Program, six months after its conclusion.	A randomized controlled community trial was carried out in two centers of the “Academia da Saúde” Program, randomly identified as control or intervention groups. The study involved 291 adults and seniors	Six months after the end of the intervention, the beneficial results obtained for physical activities and nutritional status were not maintained. The benefits related to eating habits remained, but not exclusively due to the effect of the intervention.

			involved in the routine activities of the “Academia da Saúde” Program. Individuals in the intervention group also participated in the Active Life Improving Health Program for 12 weeks. Accelerometers were used to assess physical activity, questionnaires to assess eating habits and anthropometric measures for nutritional status.	
**Nutrition and Anthropometric Profile**				
<u>***Users, n = 03***</u>				
	[50]	To evaluate the impact of the intervention on the dietary and anthropometric profile of users of the “Academia da Saúde” Program.	The nutritional intervention, which lasted 11 months, consisted of food and nutrition education groups. The impact of the intervention was measured by comparing dietary and anthropometric indicators of two	Users who participated in less than 50% (n = 61) of the nutritional intervention showed a reduction in the daily consumption of sweetened soda (p = 0.03), and those who participated in 50% or more (n = 63) reduced consumption daily per capita oil (p = 0.01) and sugar

		groups divided according to the percentage of participation in the intervention.	(p = 0.002); increased consumption of fruits (p = 0.004) and milk and derivatives (p = 0.02), showing weight reduction (-1.3 ± 3.9 kg; p = 0.02).
[36]	To characterize the consumption of micronutrients by users of “Academia da Saúde” Program and verify its possible association with nutritional status.	Anthropometric measurements were evaluated that allowed the calculation of the Body Mass Index and waist-to-hip ratio. Dietary intake was assessed using two 24-hour recalls on non-consecutive days, obtaining mean values for vitamins A, C and D and the minerals zinc and calcium.	High inadequacy of micronutrients was observed regardless of nutritional status, with emphasis on vitamin D and calcium, in addition to a higher prevalence of inadequate intake of the mineral among overweight individuals. Consumption of this vitamin D and calcium was also lower among women with high and very high waist circumference and was inversely related to waist circumference and waist-to-hip ratio. The highest consumption (highest quartile) of vitamin A was associated with a lower chance of being overweight, relating to the waist-to-hip ratio. Higher zinc intake (third and fourth quartiles) was associated with

			greater odds of being overweight.
[37]	To evaluate the impact of a nutritional intervention based on the Transtheoretical Model for weight control in overweight women, users of the "Academia da Saúde" Program according to food consumption, anthropometric and biochemical parameters.	The control group participated in the service's routine activities (physical exercise and collective food and nutrition education actions) and those in the intervention group received individual nutritional counseling, based on the Transtheoretical Model, for six months. In addition, the sociodemographic, health and dietary profile was investigated, including the stages of change for weight control and anthropometric characteristics. A random subsample (40%) was evaluated for metabolic and inflammatory parameters (total cholesterol and fractions,	The nutritional intervention based on the Transtheoretical Model for weight control was effective in reducing body weight, as well as improvements in the health and nutrition profile of the participants.

		triglycerides, fasting blood glucose, plasma insulin, adiponectin, resistin, IL-6, IL-10 and TNF-)	
	**Physical Activity**		
Physical Education Professionals, n =02			
[64]	To understand the knowledge that is mobilized for the work carried out by Physical Education professionals developed in the “Academia da Saúde” Program.	Recorded, transcribed and analyzed interviews using Content Analysis were used.	In-service training, continuing education and the use of informal tutors in service stood out as sources of reference for the mobilization, construction and re-signification of the knowledge necessary for the performance of the Physical Education professional in the “Academia da Saúde” Program. Knowledge from experience, procedural knowledge, knowledge from professional training and even disciplinary knowledge constitute a “tool-knowledge box” that can be constituted from initial training and continued in live work in action.

[65]	To analyze the knowledge constituted and mobilized by physical education professionals who work in the "Academia da Saúde" Program of the Macromissionary Region of the State of Rio Grande do Sul and its relationship with context requirement	Data were produced between December/2015 and May/2016 from the information systems of the Ministry of Health, in particular CNES and TABNET. Additionally, semi-structured interviews were conducted with 6 physical education professionals who develop their activities in the "Academia da Saúde" Program in the chosen region.	The performance of Physical Education Professionals stems from the demands of the context in which they are inserted. The context, in turn, instigates the mobilization of knowledge from professional and experiential training to organize and execute the actions of the "Academia da Saúde" Program. The context contributes in certain cases for professionals to constitute and mobilize procedural knowledge, especially with regard to the dynamics of the service, which allow some integration of the program with the other health services offered, although in a fragmented way. The work of physical education professionals is strongly linked to the practical dimension of activities and is legitimized from a primarily biological and preventive discourse, little aligned

				with the principles of basic health care. Professionals mention the importance of dialogue in their interventions, which constitutes experiential knowledge. However, the dialogue does not result in actions aimed at promoting health from the perspective of autonomy and empowerment.
<u>Users, n = 05</u>				
	[66]	To understand the meaning of physical activity and the “Academia da Saúde” Program for its users and discuss the consequences of the Previne Brasil Program on the structure and functioning of the program.	Supported by the theory of social representations, 33 in-depth interviews were carried out with users of the “Academia da Saúde” Program in Belo Horizonte, interpreted by Structural Narration Analysis.	It was revealed that there are profound representations about physical activity as a remedy for physical, psychosocial and other illnesses, as a contribution to improving the quality of life. Users see the “Academia da Saúde” Program as a necessary and inclusive health promotion policy, as it offers free access to quality physical activity, defined as one of the main aspects for adherence. The planned funding of the Previne Brasil Program

			does not include specific funding for maintaining the team of the Expanded Family Health Center in Primary Care, removing the responsibility of municipal managers to assemble multidisciplinary teams. The absence of these teams, which include physical education professionals, responsible for 90% of the actions of the “Academia da Saúde” Program, may compromise the results recognized by program users, especially those related to the reduction of access inequalities and also the various promotion actions of health in Primary Care.
[41]	To analyze the relationship between the presence or absence of the “Academia da Saúde” Program on the population's level of	Data from the Risk Factor Surveillance system and Protection for Chronic Diseases by Telephone Inquiry (VIGITEL) between 2006 and	In analyzes adjusted for year, gender, age and education, the chances of achieving sufficient levels of physical activity during leisure time were 1.04 (95%CI: 1.00-1.08) times greater among exposed

	physical activity during leisure time.	2016.	individuals. The odds of achieving sufficient leisure-time physical activity levels were 1.09 (95%CI: 1.04-1.15) times greater among exposed women. since 2011 compared to a control group of unexposed women. No other statistically significant results were found. It was concluded that PAS cannot substantially affect entire populations.
[77]	To understand the perception of users about the "Academia da Saúde" Program, with emphasis on structure, functioning and professional support in health promotion practices.	Data were collected through interviews and synthesized through thematic analysis.	There was a lack of qualified professionals to assist with the exercise and to provide the necessary guidance regarding the practice of activities. It was also noted that the distance between the residence and the center can be a potential incentive or a disincentive. The location of centers far from homes are obstacles for the population to routinely adhere to physical activities.
[58]	To analyze the correlation	Adherence indicators (IND-	The IND-ADE was higher in

	between municipalities' adherence to the “Academia da Saúde” Program, hospitalizations for chronic noncommunicable diseases and socioeconomic levels, in the period 2011-2017.	ADE) were calculated for 2,837 Brazilian municipalities to the “Academia da Saúde” Program, and hospitalizations due to non-transmissible chronic diseases - NCDs, according to financing categories and the Firjan Socioeconomic Development Index.	municipalities funded by parliamentary amendments (1.18), with moderate to high IFDM (0.94) and high IND-NCDs (1.03) (p<0.001). There was a positive correlation (p<0.05) between IND-ADE and IND-NCDs in municipalities covered by resources from the Ministry of Health (r = 0.14) and from both types, parliamentary amendments and ministerial program (r=0, 12), and negative correlation in municipalities with moderate to low Firjan Socioeconomic Development Index (r=-0.09; p=0.013).
[59]	To evaluate the impact of the “Academia da Saúde” Program on mortality from systemic arterial hypertension in the state of Pernambuco, Brazil.	Socioeconomic, demographic and epidemiological data from the 89 municipalities that implemented the program (treated) and another 52 that did not (controls) in the years 2010	Treated counties had an overall 12.8% decrease in the hypertension mortality rate, from 12.5% among people of brown color and 13.1% among people over 80 years of age.

		and 2017, from the Department of Informatics of the SUS, the Brazilian Institute of Geography and Statistics and other databases were analyzed by applying the Propensity Score Matching method.	
**Program assessment**			
<u>"Academia da Saúde" Program , n = 08</u>			
[67]	To understand which imaginary body the government proposes for the subject.	Ordinance No. 2681 of November 7, 2013, which regulates public action and images of the physical space of Academia da Saúde, was used as material for analysis.	It was found that the act of attributing responsibility for their health to the subject is recurrent in this policy. In this way, the function and duty of the State to ensure the well-being of the population are not considered. Still, the "Academia da Saúde" Program appears not as a duty, but as a benefit of the public power for the subject.
[79]	To assess the evaluability of the Academia da Saúde	Descriptive and exploratory analysis of the operational	It was found that managers they have little knowledge about the program's

	Program in Recife	aspects of the program's actions and context, using the seven-element framework system, which allowed for the description and development of logical and theoretical evaluation models, in addition to identify evaluative questions.	guidelines and that the official documents present a wide range of objectives, principles and guidelines, without, however, identifying the goals for the program to fulfill its purpose.
[68]	To analyze whether and how the “Academia da Saúde” Program is based on the principles and guidelines of the health promotion (comprehensiveness, intersectoriality, empowerment, participation and territory).	Ordinances and manuals of the Ministry of Health, of public access, published between 2011 and 2015 were evaluated. The categories analyzed were: integrality, intersectoriality, empowerment, participation and territory.	It was identified in the research that the “Academia da Saúde” Program presents itself as a health promotion program due to its approximation with the assumptions of health promotion: integrality, participation, empowerment, intersectoriality and territory, and demonstrates an approximation of the program with the selected theoretical framework. The description of the design of the “Academia da Saúde” Program advances towards breaking with a model

			of health care program focused on the disease or on a specific professional category in search of comprehensive care. The “Academia da Saúde” Program is a national program that considers the context and local culture of the territory from its design, as well as social participation. Still, it has great potential for impact on the population, in the reorientation of professional and care practices, in addition to instigating social mobilization through the creation of skills to improve living conditions.
[69]	To evaluate the quality of the current Form Poles in Operation, used in the monitoring of the “Academia da Saúde” Program.	Data were collected through a checklist, an evaluation instrument capable of identifying problems and signaling existing difficulties in products and programs. The checklist was constructed from	The utility standard of the Operating Poles Form was partially met, while in the adequacy and accuracy categories the standards were not met, demonstrating that the instrument must be improved for a better monitoring of the Program.

		congruence analysis and technical validation of the adapted description of the 26 items of selected standards in the categories of usefulness, adequacy and accuracy of the Joint Committee, having its technical and content validity evaluated by specialists.	
[70]	To analyze how the “Academia da Saúde” Program constitutes an element of Health Promotion and Education in the SUS: 1) identify, describe and verify its links constitutive; 2) understand and examine the documents referring to the bases of its creation and	Documental research of public health policies and ordinances about the “Academia da Saúde” Program and a bibliographical research in databases, magazine articles, database of dissertations and theses, among others.	Health Promotion actions lack educational practices based on the Collective Health paradigm, so as not to reproduce health education activities based on the biomedical paradigm in the “Academia da Saúde” Program. Health education in the “Academia da Saúde” Program has its theoretical bases based on popular education, which is fundamental in the consolidation process of the Unified Health System.

	structuring; 3) carry out the mapping and description of the current conditions of its implementation in the country; 4) and analyze its theoretical bases, identifying its mediations with education.		
[80]	To analyze the distribution of municipal adhesions to the “Academia da Saúde” Program in the national territory and the impact of changing the type of financing for works on the number of adhesions.	Secondary data provided by the Ministry of Health regarding adherences that occurred from 2011 to 2017 were used to compose the study variables.	Most macro-regions had more than 50% of municipalities participating in the “Academia da Saúde” Program. The Northeast and Southeast regions had more adhesions, but the North region stood out with the highest number of municipalities – “Academia da Saúde” Program in proportion to the total number of municipalities. The period of greatest expansion was from 2011 to 2013. The number of adhesions and municipalities to the “Academia da

			Saúde” Program in the five years of exclusive funding by parliamentary amendment did not exceed the amount approved from 2011 to 2012. In the first years of funding by parliamentary amendment, there was a concentration of adhesions in the same municipalities. All macro-regions had more than 60% of completed works, however the percentage of gyms with federal funding among the adhesions eligible for receipt was low.
[91]	To evaluate the degree of implementation of the “Academia da Saúde” Program in the city of Bezerros, Pernambuco.	The study was divided into four stages: i) validation of a logical model for the program; ii) development of indicators and judgment matrices; iii) validation of matrices by specialists, and; iv) verification of the degree of implementation	The degree of implementation of the “Academia da Saúde” Program in Bezerros was considered intermediate (55.0%), with the structure dimension having a higher score (64.7%) than the work process (48.6%).

		through interviews. A scoring system was used to describe the degree of implementation as: 1) Incipient: 0 > 33.3%; 2) Intermediate: > 33.3 < 66.6%; 3) Advanced: > 66.6%.	
[81]	To analyze the degree of implementation of the Academia da Saúde Program in a medium-sized Brazilian city, Vitória de Santo Antão, Pernambuco, in 2018.	Evaluative study that carried out a normative approach, considering the dimensions of structure and process related to the work process. The research was divided into: (1) elaboration of the theoretical evaluation model, through validation of the logical model; (2) verification of the level of implementation, through interviews with workers and managers.	The level of implementation was considered intermediate (37.54%). The structure score was higher (54.76%) than the process score (26.06%).
<u>Physical Education Professionals, n = 03</u>			
[71]	To know the characteristics	Questions about the existence or	The work processes were characterized

of Physical Education Professionals who work in the Academia da Saúde program in the Metropolitan Region of Recife, and to identify the difficulties faced in their work process.

not of difficulties in the work process, in addition to questions related to: a) inputs for carrying out the work and evaluating actions; b) articulation of the work at the center with the Primary Care teams; c) articulation with the coordination of the program for the planning, execution and evaluation of the actions were evaluated. The questionnaire included sociodemographic, training and hiring variables (age, gender, time since graduation, ownership, participation in disciplines or internships in the area of public health during graduation; form of hiring and type of link with

by the precariousness of the material used in the gym classes, inefficient participation of the program's coordination in the planning, execution and evaluation of the actions and difficulties in articulating the program's activities to the other actions and services in the Primary Care network.

		the program) .	
[72]	To know the perception of Physical Education professionals about the objective of their activities, the object of their professional intervention and the products of their work in the "Academia da Saúde" Program.	Data collection involved a semi-structured interview. The interview contained questions about: i) the objective of the professionals' work in the Academia da Saúde Program; ii) the means to achieve these objectives; iii) the products generated by this work; iv) how the work of professionals contributes to the Family Health Strategy, and; v) the professionals' perception of health promotion.	Physical Education Professionals identified health promotion as the objective of their actions and individuals and communities as objects of their intervention, in addition to describing the improvement of physical/functional performance, social interaction and biopsychosocial benefits as the main products of the work in the program. On the other hand, it turns out that the perception of the concept of health promotion is focused on changing behaviors, and that the lack of articulation of the program's actions with the Primary Care teams can compromise the reach of interdisciplinarity and the organization of multidisciplinary work processes with a view to comprehensive care in the Unified Health System.

[73]	To understand the representations of “Academia da Saúde” Program professionals about health/disease, which underlie health promotion activities, aimed at the population.	Based on the theory of Social Representations, understanding them as points of view, made explicit in human action, in-depth interviews with physical education professionals from the “Academia da Saúde” Program in Belo Horizonte were interpreted by the Structural Analysis of Narration.	It was found that there are central representations that define health as the opposite of disease, peripheral representations, with health being more than the absence of disease. The present biomedical model coexists with points of view that expand health practice, focused on building health promotion as an asset and a right. To keep these representations parallel, professionals use, contradictorily, the official discourse that created the Program, anchored in Collective Health, and the discourse of science and common sense, anchored in Biology. The professional's involvement in actions becomes paradoxical, requiring improvements in their training to continue the paradigm shift in health care.

Managers and Professionals, n = 03

[82]	To analyze the "Academia da Saúde" Program in municipalities in the state of São Paulo in relation to the policy cycle (entry into the agenda, training and implementation) and to identify the limits of insertion in the health care network and in the National Programs of Primary Care and the National Program of Health Promotion.	For the analysis referring to the policy cycle, data were extracted through bibliographic review and document analysis. As for the empirical case study, aiming to identify the limits of the program, semi-structured interviews were carried out with managers and professionals. The analysis was carried out based on the dimensions: political and historical context, organizational model and management models of the poles through successive approximations.	The induction process was accentuated by the little presence of the state government in decision-making and implementation of federal programs. State involvement took place in a formative manner. Implementation projects are non-specific, bringing little data about their action proposal, integration with the health care network, more specifically Primary Care, and with the intersectoral community support network. The articulation with the assistance network occurs in an irregular and fragile way. The lack of knowledge of the objectives on the part of professionals and managers, which hindered working relationships. Despite belonging to the care network, the program is contextually displaced, since there is no integration between this

			service and others. Also, the services are performed separately by each professional and by each health equipment.
[90]	To measure the evaluability of the "Academia da Saúde" Program in the city of Recife, considering the description of the intervention, the elaboration of its logical and theoretical evaluation models, in addition to identifying evaluative questions.	Qualitative approach based on the system of seven elements, through documentary research, interviews and discussion with managers and technicians of the program and the technique of condensation of meanings for analysis of documents and interviews.	It was found that the program has a wide range of objectives, principles and guidelines, and that some of the managers are unaware of these elements. After modeling the program, the methodological and operational viability for the development of an evaluative research was observed, which was discussed and agreed with managers and workers.
[76]	To identify the perception of managers of physical activity programs about the barriers to the functioning	Information was obtained through a questionnaire developed to evaluate interventions to promote	The main barriers were related to lack of human resources, lack of incentive from management, lack of material conditions, facilities, equipment and lack of

	of the “Academia da Saúde” Program centers in Santa Catarina.	physical activity in Primary Health Care.	standardized instruments to carry out the evaluation of actions.
<u>Managers, n = 04</u>			
[83]	To describe the scenario of the implementation of the Program and present characteristics of its operation in the country.	Data were collected using an electronic form sent to all Municipal Health Departments that received funds to implement the Program.	A total of 856 poles reported being in operation, primarily developing body practices, healthy eating and health education activities. The main public participating in the Program were adults and the elderly. Difficulties pointed out by managers include the inclusion of children and adolescents and the hiring of professionals. More than 90% of the centers do not depend exclusively on federal resources for their operation, receiving municipal contributions for the development of their activities.
[60]	To describe the health promotion actions that were planned in the	A telephone survey was carried out with 914 health secretaries from the municipalities that	The actions would reach more than one million people in 25% of the municipalities. Improvements in physical

	municipalities that received resources to develop the "Academia da Saúde" Program.	received funds until July/2012.	activity and healthy eating are priority actions. Most cities had partners for actions. Only 50% of the municipalities used health indicators for planning the program and 25% of the municipalities planned training for health professionals and the management group to coordinate the program. The actions focused on physical activity and healthy eating. A large number of people would be reached in actions. Only half of the municipalities used health indicators for the plan and few municipalities planned professional training and a management group.
[84]	To analyze the federative relationships involved in the implementation of the "Academia da Saúde" Program in municipalities in the state of São Paulo.	Document analysis and semi-structured interviews were used. Additionally, the theoretical framework of the policy cycle was used. The cycle subdivides the political process into five	Direct induction by the Ministry of Health in the municipality was observed through standardization, political interests and financial incentives. The participation of the states is bureaucratic and restricted to the Bipartite Inter-

		phases: (1) setting the agenda; (2) policy formulation; (3) decision making; (4) policy implementation and (5) policy evaluation.	management Commission. Municipalities are financially dependent for implementation. There is no concrete possibility of adapting to local realities.
[85]	To analyze the implementation of the “Academia da Saúde” Program over three years (2015 to 2017).	The data used came from the annual monitoring of the implementation of the “Academia da Saúde” Program in Brazil, through questionnaires answered by state, municipal and center management.	There was an increase in the monitoring response rate, especially in the northeast region. More than 88% of the poles maintained a greater proportion of physical education professionals. There was a significant increase in the offer of body practices and physical activity (2015 vs 2016) and a reduction in actions in food (2015 vs 2016), complementary integrative practices (2015 vs 2016) and care for the elderly (2015 vs 2017).
<u>Managers, Professionals and Users, n = 06</u>			
[86]	To analyze the implementation of the National Health Promotion	A qualitative and quantitative approach was combined. In view of the study design (exploratory-	The preventive and functionalist character inscribed in the ordinance of the institution of the Academia da Saúde

Policy, carried out through the “Academia da Saúde” Program, and its configuration in the micro-spatial context: 1) access and participation; 2) understanding and incorporation of beliefs and values about health promotion in the “Academia da Saúde” Program.

descriptive), multiple research and data collection methods and techniques were employed. Thus, both documentary research and descriptive and field statistics were used.

Program, by proposing as a central objective the promotion of bodily practices, physical activities, leisure and healthy lifestyles, is presented in the realities of the subjects and in the territories of Goiás. Therefore, even admittedly being a free access program and for the general population, the presence of the elderly population and/or people affected by non-communicable chronic diseases, such as hypertension and/or diabetes, is observed. The users' participation was restricted to their presence in the activities developed, not presenting processes that formalize and encourage their actions in the management of the program. This is also demonstrated in the data extracted from the monitoring, which showed the low representation of these users and family

			members in the management support group.
[74]	To analyze the perception of users, managers and health workers about the operability, the actions developed at the “Academia da Saúde” poles and their integration with the other points of the Health Care Network (RAS)	Semi-structured interviews were carried out with the study participants and data analysis was based on a methodological and analytical proposition based on critical hermeneutics.	The “Academia da Saúde” Program demonstrated positive influences on the practice of physical activity of the user population and, consequently, improvements in health conditions and quality of life. The results identified the functioning dynamics and organization of the activities, highlighting: the facilities and difficulties regarding its operability; the actions frequently developed; the advances observed in relation to health promotion and quality of life; and comprehensiveness in the “Academia da Saúde” Program.
[87]	To evaluate the performance in a pole of the “Academia a Saúde” Program in Belo	Through qualitative, descriptive research, of the case study type, an in-depth interview was adopted for data collection. The	The results indicated some gaps in the program, such as adequate logistical aspects, inadequacy of the articulation between the center and the Basic Health

	Horizonte, Minas Gerais.	sample was stipulated by the saturation technique.	Unit, and the absence of defined goals. As positive aspects, the professionals' satisfaction in working in the program and the students' satisfaction in participating in the classes were identified.
[75]	To analyze the pedagogical process developed in face-to-face training (based on the syllabus of the program's technical notebook and aimed at building technical capacities related to program management) applied in the state of Tocantins.	Educational research of a qualitative nature, of the action-research type, in which content analysis and document analysis techniques were used for data analysis.	The analysis of the construction process of the technical notebook revealed an awareness-raising and dialogical methodology with fundamental contributions to the enrichment and relevance of the contentes programmatic issues covered in face-to-face training. Regarding the construction of the pedagogical process of face-to-face training, the findings demonstrated a dialectical and dialogic movement on the part of the responsible educational team. Regarding the teaching-learning process, pedagogical trends aligned with the

			theoretical-methodological framework proposed by educator Paulo Freire were identified.
[61]	To analyze the work of health promotion in a unit of the “Academia da Saúde” Program, in the city of Lajeado, Brazil.	Data were collected through condensed fieldwork, involving interviews and non-participant observation of group sessions.	Using the theory of salutogenesis, the qualitative analysis generated results on the elements of health promotion work, namely: holistic care, use of diversification in cognitive, psychosocial and physical activities, development of skills and abilities that served as health resources , and the occurrence of resistance and challenges in the program. In addition, the results offered practical examples of possibilities to mobilize the health promotion process, which represents a significant contribution to knowledge about health work.
[88]	To analyze actions to encourage physical activity within the scope of the	A qualitative approach was used. Multiple research and data collection methods and	The implementation of the “Academia da Saúde” Program did not take place in Goiânia and physical activity is not being

	health promotion policy in the municipality of Goiânia-Goiás.	techniques were employed. In this way, both the application of a questionnaire, an observational diary and documental research were used. The questions included information about the implementation of the Health Promotion Policy, as well as aspects related to the quality of life and activities carried out by users.	taught by Physical Education professionals from the Family Health Support Center (NASF). The non-perception of potential users in relation to the NASF as a nucleus composed of a multidisciplinary health team stands out. The study's conclusions also indicate that the main impediment to the implementation of the Health Academy Program is in the administrative and political procedures, since the resource for its implementation has been made available.
Users and Territory, n = 01			
[56]	To verify the validity of secondary data in the investigation of the food environment and to analyze the characteristics of the community and consumer	Validation of establishments that sell fruits and vegetables, obtained using public databases, was carried out by telephone contact, use of the Google Street View tool and audit. The	The audit revealed weak agreement (45.7%) of the secondary databases. Of the 298 establishments audited, most were grocery stores and street markets (61.3%), which had a greater availability of healthy foods, but also significantly

		environment in health promotion service territories.	investigated community food environment variables were: type of establishment and location; and the consumer's environment: availability, variety, price and advertising of fruits and vegetables, and availability and variety of ultra-processed foods; and hygienic-sanitary aspects. To measure access to healthy foods, the access index to these foods was used.	sold ultra-processed foods (60.7%). As for sanitary conditions, 1/3 of the establishments were disapproved.
<u>Users, n = 02</u>				
	[62]	To carry out an analysis of the assistance provided to the elderly, in basic health care, in view of the implementation of the Academia da Saúde	Two cross-sections, in two periods separately, namely the years 2012 and 2014, which correspond to the two cycles of the National Program for the Improvement of Access and	There was an improvement in the standard of assistance to users, with 2014 showing better results than 2012, in almost all evaluation dimensions for Primary Care, except for some aspects of the dimension “Assistance to users with

	Program, based on the National Program for the Improvement of Access and Quality of Care Basic.	Quality of Primary Care were evaluated, and the database from the external evaluation phase was used as a source of information.	Diabetes and Systemic Arterial Hypertension", which it worsened in 2014. Although there was no association between the municipality having centers of the Academia da Saúde Program and the improvement in health conditions, the increase in the implementation of these centers in the country during the investigated period is notorious.
[38]	To validate the self-reported body weight of users of the "Academia da Saúde" Program, Belo Horizonte, Minas Gerais, and verify factors associated with weight error.	Self-reported body weight, obtained by telephone interview, was compared to the measured weight.	Men validly reported their weight (error=0; p=0.15). Agreement for overweight classification was 94.3% (Kappa=0.88). Overweight women aged >30 years had a greater error in reporting (-0.8kg; p<0.01). After correcting for multiple regression, the validity of the self-reported was satisfactory (error=0; p=0.99).

Instrument assessment at local level

“Academia da Saúde” Program, n = 01

	[39]	To evaluate the relative validity of methods of investigation of the consumption of fruits and vegetables by users of the "Academia da Saúde" Program in Belo Horizonte, Minas Gerais.	The medians of consumption in grams of fruits and vegetables (except tubers), tertiles and adequacy of consumption of these foods according to test and reference methods were compared.	Better correlations were observed for the evaluation of fruit consumption according to QBreve-FH (r=0.437), mainly among individuals with higher education (r=0.521) and overweight (r=0.551). No statistical differences were observed between consumption in grams of fruit obtained by QBreve-FH and R24h. This comparison also resulted in higher percentages of classification in exact tertiles (46.1%). All test methods overestimated the consumption of vegetables and fruits and vegetables when added together. They also showed poor correlation when the adequacy of consumption of these foods in grams was analyzed.
**Health education**				
Users, n = 01				
	[78]	To understand	The Theory of Social	Despite persistent hegemonic

		representations about health and illness of users of “Academia da Saúde” in Belo Horizonte, Minas Gerais.	Representations, from the perspective of Alain Giami, supported the study, carried out from interviews. The data were interpreted based on the Structural Analysis of the Narration.	representations, health academies are presented as a space for construction and possibilities for ruptures, indicating that professionals at academies must increasingly commit to expanding and deepening the understanding of broader concepts about health , disease and care, considering the importance of psychosocial aspects in investing in healthy lifestyles. The offer of actions must prioritize autonomy, completeness, equity, based on the protagonism of users over their choices.
**Description and Assessment of the Program at the local level**				
Users, n = 02				
	[63]	To analyze indicators related to the care of adolescent users in programs and interventions to promote physical	Indicators related to user service were obtained by applying four instruments: questionnaires in the manager version, professional version, user	Pernambuco municipalities offered at least one program/intervention to promote physical activity as part of health services for adolescents. However, the number of adolescents assisted in

	activity that are developed in Primary Health Care in the state of Pernambuco.	version and environment observation script. Variable analysis included descriptive and inferential statistical procedures.	these services was low, and both the activities developed and the available infrastructure were not aimed at attracting this population subgroup.
[89]	To describe the history and evaluation methodology of the “Academia da Saúde” Program in Belo Horizonte, MG and discuss it as an experience in promoting health and equity.	Perception of quality of life, satisfaction with life, social life and participation in the neighborhood were compared only between female users and non-users aged 40 years and over.	Better perception of quality of life, positive life satisfaction, greater reports of social interaction and participation in the neighborhood were observed among women with higher income compared to their counterparts ($p < 0.001$). On the contrary, users reported better perception for all the evaluated constructs in relation to lower income peers.
**Methodological study**			
Users, n = 01			
[51]	To present the protocol of the randomized controlled community trial that was conducted to encourage the consumption of fruits and	Consumption of fruits and vegetables was classified according to the stages of change in the transtheoretical model. The educational activities	The intervention applied based on the chosen theories contributed to the refinement of the intervention through the inclusion of different educational strategies, which considered the context

	vegetables, a community educational nutritional intervention conducted to encourage the consumption of fruits and vegetables, carried out in primary health care in Brazil.	were based on the transtheoretical model and on the problematizing-dialogical pedagogy, planned by an interdisciplinary team. The actions were carried out over seven months. Educational strategies included workshops interspersed with motivational messages conveyed through postcards, interactive activities based on the environment and informative material.	involved in the investigated objective. But even so, the intervention proved to be viable for large population groups and for the health services scenario.
		**Healthy lifestyle**	
<u>Users, n = 01</u>			
[40]	To measure healthy lifestyles according to the time of participation in the “Academia da Saúde” Program.	Data on healthy lifestyles collected included daily fruit and vegetable intake, physical activity, body mass index, smoking, and alcohol-related	Almost half of the participants (43.3%) had three healthy lifestyle factors. The prevalence of having all five factors varied according to the time of participation in the service. The lowest

			habits. The time of participation in the "Academia da Saúde" Program was calculated by the difference between the date of enrollment in the program and the date of data collection.	rates were found in the first quartile (4.9%) and the highest rates in the fourth quartile (8.1%). Those who attended the service for a longer time (fourth quartile) were more likely to have a healthy body mass index (OR = 1.43; 95%CI: 1.14-1.80; p = 0.002) and to avoid smoking (OR = 1.62; 95% CI: 1.06-4.49; p = 0.01), in relation to those who attended the program for less time (first quartile).
**Program assessment conducted in the context of the "Academia da Saúde" Program**				
<u>Users, n = 02</u>				
	[52]	To evaluate the effectiveness of the Vida Ativa Melhorando a Saúde (VAMOS) program based on the perception of users of the "Academia da Saúde" Program in Belo Horizonte, Minas Gerais.	Effectiveness was assessed through reports in three focus groups using 'the script based on the dimensions of the RE-AIM model.	From the content analysis, benefits were identified in four categories: physical activity, diet, body weight and quality of life.

[53]	To evaluate the biopsychosocial impact on the symptomatology of illnesses of participants with schizophrenia and/or bipolar affective disorder in a physical activity program at “Academia da Saúde”.	Data were collected from the Sociodemographic Questionnaire instruments; Diagnosis for Psychoses and Affective Disorders; Positive and Negative Syndrome Scale; Calgary Depression Scale for Schizophrenia; International Physical Activity Questionnaire; and Internalized Stigma Scale.	The program showed positive effects on the parameters evaluated, such as a decrease in psychosis, related to physical and mental well-being, with a decrease in stress, depression and improvement in mood, self-esteem, disposition, in addition to minimizing stigma.
**Reduction of health expenses**			
Users, n = 01			
[43]	To evaluate the impact of the “Academia da Saúde” Program on spending on hospital admissions for cerebrovascular diseases.	Socioeconomic, demographic and epidemiological data from 89 municipalities that implemented the program (treated) and another 52 that did not (controls) were used. Data were obtained from the Department of Informatics of the	The municipalities that implemented the "Academia da Saúde" Program spent an average of R$ 1,258.61 less on hospitalizations for cerebrovascular diseases ($p < 0.05$) for each group of 10,000 inhabitants.

		Unified Health System, Brazilian Institute of Geography and Statistics and other databases.	

IG: Intervention Group; CG: Control Group; NASF: Family Health Support Center.

Supplementary Table 2. Descriptive characteristics of the included studies.

**Variables**	**n**	**%**
Place where the studies were carried out		
Brazil	10	13.5
Minas Gerais (Brazilian state)	40	54.4
Pernambuco (Brazilian state)	09	12.2
São Paulo (Brazilian state)	02	2.7
Rio Grande do Sul (Brazilian state)	02	2.7
Espírito Santo (Brazilian state)	01	1.3
Santa Catarina (Brazilian state)	01	1.3
Goiás (Brazilian state)	02	2.7
Ceará (Brazilian state)	02	2.7
Tocantins (Brazilian state)	01	1.3
Mato Grosso do Sul (Brazilian state)	01	1.3
Mato Grosso (Brazilian state)	01	1.3
Two or more Brazilian states	01	1.3
Not described	01	1.3
Year of publication		
2014-2015	10	13.5
2016-2017	20	27.0
2018-2019	20	27.0
2020-2022	24	32.5
Number of participants		
0-99	23	31.0
100-499	07	9.4
500-999	03	4.1
1000-1499	03	4.1
≥ 1500	19	25.7
Not described/does not apply	19	25.7
Nature of studies/documents		

Scientific article	54	73.0
Dissertation	19	25.7
Thesis	01	1.3
Design of studies/documents		
Cross-sectional	48	64.9
Ecological	05	6.7
Case study	09	12.2
Intervention	09	12.2
Documentary	03	4.0
Information analysis		
Quantitative	45	60.8
Qualitative	25	33.8
Quantitative and Qualitative	04	5.4

IG: Intervention Group; CG: Control Group; NASF: Family Health Support Center.
